# Usefulness of High-Frequency Ultrasonography in the Diagnosis of Melanoma: Mini Review

**DOI:** 10.3389/fonc.2021.673026

**Published:** 2021-06-11

**Authors:** Maria Paola Belfiore, Alfonso Reginelli, Anna Russo, Gaetano Maria Russo, Maria Paola Rocco, Elvira Moscarella, Marilina Ferrante, Antonello Sica, Roberto Grassi, Salvatore Cappabianca

**Affiliations:** ^1^ Department of Precision Medicine, University of Campania Luigi Vanvitelli, Naples, Italy; ^2^ Dermatology Unit, University of Campania Luigi Vanvitelli, Naples, Italy; ^3^ Italian Society of Medical Radiology (SIRM) Foundation, Milan, Italy

**Keywords:** Melanoma, high frequency ultrasound, oncology research and diseases, MDT, Dermatology

## Abstract

High-frequency equipment is characterized by ultrasound probes with frequencies of over 10 MHz. At higher frequencies, the wavelength decreases, which determines a lower penetration of the ultrasound beam so as to offer a better evaluation of the surface structures. This explains the growing interest in ultrasound in dermatology. This review examines the state of the art of high-frequency ultrasound (HFUS) in the assessment of skin cancer to ensure the high clinical approach and provide the best standard of evidence on which to base clinical and policy decisions.

## Introduction

Cutaneous melanoma (CM) has a high incidence rate, even among young people; it has steadily increased over the last several decades (1, 2). Moreover this incidence is 1.5 times higher in males (3). However, this data is related to the age of onset; it has been seen that melanoma affects young women and older men. The main risky factors implicated in melanoma development are exposure to ultraviolet (UV) for their genotoxic effect, the number of melanocytic nevi, familiar history, and genetic susceptibility (3). It has been noted that patients with a previous history of melanoma have a 1% to 8% risk of developing other primary melanomas (4). These numbers highlight the health and socio-economic implications of this skin cancer. Melanoma is related to a poor prognosis in the general population. The main important prognostic factors for survival are the Breslow’s index and the presence of ulceration. In the eighth edition, the AJCC melanoma expert panel described the impact of the tumor thickness subcategorizing T1 melanomas (5). The main prognostic factors for survival are still primary tumor (Breslow) thickness and ulceration. They are also useful to define T-category strata in cutaneous melanoma. As in prior editions, also in the eighth edition, tumor thickness has to be measured to the nearest 0.1 mm, not 0.01 mm. In this edition, melanoma thickness threshold of 1.0, 2.0, and 4.0 mm continues to define the T category. Consequently, those tumors that measure from 0.95 to 1.04 would be rounded to 1.0 mm. While in the seventh edition, a subset of these melanomas measuring 1.01 to 1.04 would have been staged as T2 (a: w/o ulceration, b: with ulceration). The clinical implication, if any, of this small group of patients who are mentioned in the eighth edition, has not yet been formally explored. Previous studies have detected a clinically significant treshold in the region of 0.7 to 0.8 mm in patients with T1 melanoma. In the eighth edition AJCC the analysis of the T1 melanoma patient cohort, multivariable analysis of factors that predict melanoma-specific survival (MSS) [i.e. tumor thickness, ulceration, mitotic rate as a dichotomous variable (<1 mitosis/mm^2^ vs ≥1 mitosis/mm^2^)] revealed that tumor thickness dichotomized as < 0.8 mm and 0.8 to 1.0 mm and ulceration could predict MSS more efficiently than mitotic rate (as a dichotomous variable).

The subcategorization of T1 melanomas (0.8 threshold) is important for the role of Sentinel Lymph nodes biopsy(SNLB) considering that SLN metastases are very infrequent (< 5%) in patients whose melanoma is < 0.8 mm in thickness and nonulcerated (i.e., AJCC eighth edition T1a) but it occurs in approximately 5% to 12% of patients with primary melanomas 0.8 to 1.0 mm in thickness. The SLN biopsy can be performed in the patients with a primary tumor thickness 0.8–1.0 mm and also in patients with thinner ulcerated tumors (i.e., all patients with AJCC eighth edition T1b melanomas). The SLN biopsy had to be performed for patients with T2 and thicker melanomas, and when performed in patients with a T1 melanoma, the status of the SLN was used (5).

**Figure 1:**
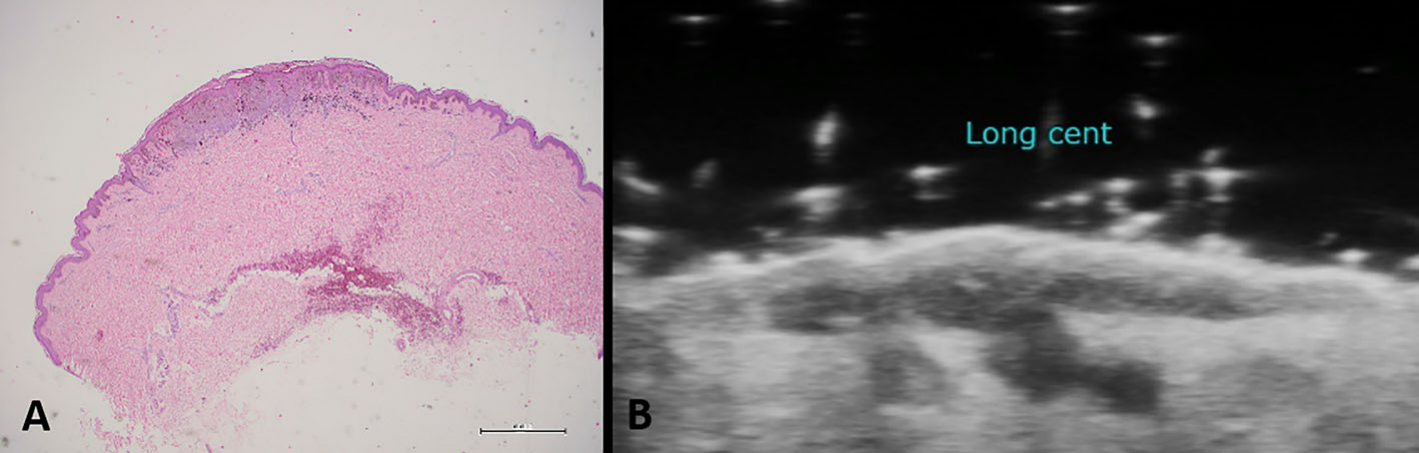
Histological specimen **(A)** and ultrasound examination **(B)** in case of cutaneous melanoma. High-frequency probes are useful for the determination of the ultrasound Breslow index, which means evaluating the depth of tumor invasion.

**Figure 2:**
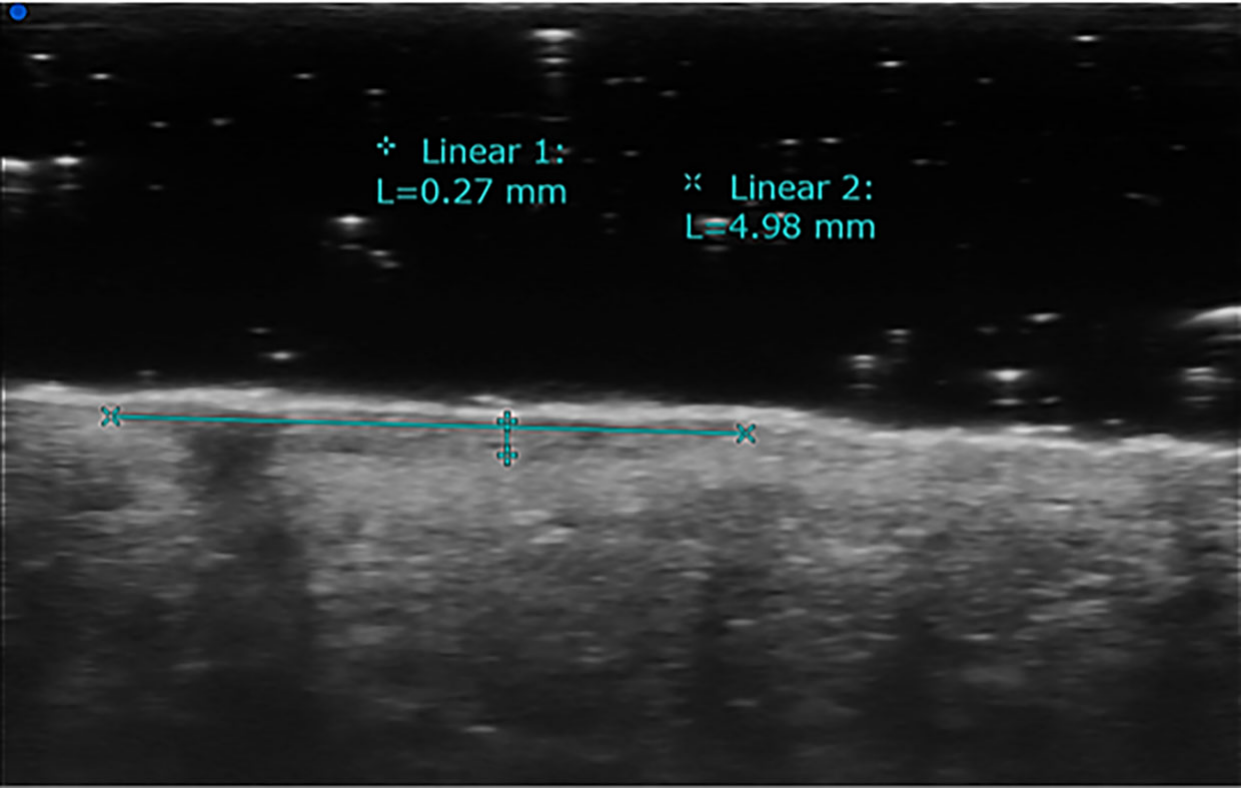
High-frequency transducers allow the determination of ultrasound Breslow index, which means evaluating the depth of tumor invasion. This example shows skin melanoma considered with HFUS (70 MHz).

The thickness of the melanoma also determines an increased risk of lymph node involvement. Patients with melanoma spread to the nearby lymph nodes have a survival rates at 5 years of 65% (6). For all patients with primary melanoma with Breslow’s index > 0.8 mm is indicated the Sentinel lymph nodes. This procedure allows the detection of metastatic involvement of the lymph nodes and the detection of nodal disease with no clinical or radiographic evidence. The outcome of SNLB may change future therapeutic management, including the choice of performing a complete lymph nodes dissection, or an adjuvant therapy, but also set up different program of clinical and imaging follow-up. For whole-body staging are used advanced imaging techniques, such as computed tomography (CT), magnetic resonance (MR), and positron emission tomography-CT (PET-CT) (7). There is no single consensus regarding surveillance imaging in melanoma patients, in fact, according to National Comprehensive Cancer Network (NCCN), the CT or PET scan is recommended every 3 to 12 months for patients with stage IIB-IV asymptomatic melanoma. While, The European Society of Medical Oncology recommends only physical examination every three months (8). However, ultrasound is the first diagnostic approach used to monitor regional lymph node basins for recurrence. It has been demonstrated that ultrasound has the highest sensitivity and specificity, 96% and 99% respectively, for lymph node surveillance (9–11), as well as for the evaluation of nodal disease. Thanks to the use of high-frequency probes, it has proved useful for the determination of ultrasound Breslow index, which means evaluating the depth of tumor invasion (**Figure 1**). Moreover, Color Doppler is an additional tool that can improve diagnostic accuracy through the identification of intra-tumor vessels and characterizations of their distributions (12) (**Figure 2**).

High accurate pre-treatment evaluation of the melanoma is useful tool for taking a correct therapeutic approach and improving the survival rate and follow-up (13).

The HFUS, and even more the ultra-HFUS, provide important information, previously obtained only thanks to biopsy samples.

**Figure 3:**
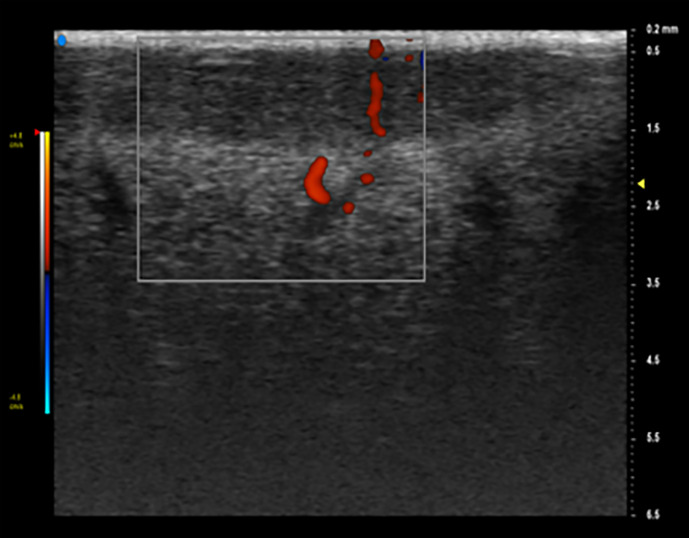
Doppler is an additional tool that can identify intra-tumor vessels and characterize their distribution, improving diagnostic accuracy. On Color Doppler examination, it is possible to see a hypoechoic lesion with an increased vascular signal.

Further information can be obtained thanks to the use of strain elastography (SE). This technique estimates the tissues elasticity according to assumption that tissues affected by tumor invasion are less deformable than normal tissues (14). An evaluation is then achieved by comparing the elasticity of the target lesion with the surrounding tissues. The data obtained on the relative stiffness is converted into a color-coded image that overlaps the two-dimensional images (15–17) (**Figure 3**).

This review examines the state of the art of HFUS in the assessment of melanoma to ensure the best clinical evaluation for the correct therapeutic strategies.

## Methods

Using the Medline, Embase, and ISI web of Science (Science Citation Index Expanded) databases, we searched different articles with these keywords: “melanoma”, “melanoma ultrasound”, “skin cancer melanoma diagnosis” (18).

The reference lists of all retrieved studies were used as additional sources of pertinent documents (18). We evaluated the title and abstract of these selected articles. If the abstract was eligible, the article was downloaded and read by two of the authors (MB and AR). We included human observational studies published from 1997 to 2020. These studies reported melanoma thickness with ultrasound (US). Furthermore, the ability to identify with HFUS the skip lesions and lymph nodes using 95% confidence intervals or other measures of statistical uncertainty. The studies included in the meta-analysis consider different epidemiological data. Many of these studies relied on specific reference incidence rates based on gender, age, and provided a relative standardized incidence ratio as risky measures (**Table 1**).

**Table 1:** List of the main studies related to the use of the HFUS in melanoma.

Author	Year	Frequency Probes	Results
Lassau et al.	1997	20 MHz	Proved that in 12 cases of melanoma the difference between histologic and US measurement was ≤ 0.2 mm.
Harland et al.	2000	20 MHz	US is a non-invasive aid for evaluating the acoustic differences between common pigmented lesions.
Clement et al.	2001	20 MHz	US is useful for differential diagnosis of skin lesions.
Bessoud et al.	2003	20 MHz	Sonographic and histologic measurement of melanoma thickness are strongly related, and US coupled with Color Doppler is a simple and useful tool for pigmented skin lesions management.
Pellacani et al.	2003	20 MHz	US measurements were slightly overestimated compared to the histological size but US has a strength correlation with melanoma thickness.
Rallan et al.	2007	20 MHz	Demonstration of quantitative differences between benign and malign skin lesions.
Gambichler et al.	2007	20 MHz	US measurements were slightly overestimated compared to the histological size but US has a strength correlation with melanoma thickness.
Machet et al.	2009	20 MHz	US measurements were slightly overestimated compared to the histological size but US has a strength correlation with melanoma thickness.
Kaikaris et al.	2011	14 MHz	They found a low US correlation between the Breslow index for thin melanomas (1-2 mm) and a significant correlation for thicker melanomas (> 2 mm).
Solivetti et al.	2014	18MHz or 22MHz (in case of very small and superficial lesions)	All of 52 lesions (in-transit metastases) were detected with HFUS.
Botar et al.	2015	40 MHz	There is not substantial difference between Breslow index and US thickness.
Reginelli et al.	2019	50-70 MHz	There is a favorable agreement between HFUS and Breslow thickness in 7 lesions examinated.

We excluded case reports, editorials, non-independent studies, and cohort or case-control studies.

**Figure 4:**
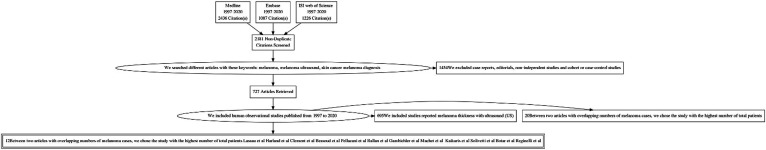
PRISMA flow diagram.

Between two articles with overlapping numbers of melanoma cases, we chose the study with the highest number of total patients (18) (**Figure 4**).

## Data Extraction

Only one co-author (MB) pulled the data into a predefined database.

The following information was considered valid for the analysis: study’s year, country, type of melanoma, number of patients, the average age, gender, and lastly, median person years accumulated by patients (18).

## Discussion

The application of new imaging techniques has also changed the staging work-up of patients with cutaneous melanoma. Chest and abdominal computed tomography (CT) scanning should be restricted to patients with high-risk melanoma (stage IIIA with a macroscopic lymph node, IIIB, IIIC) and used to evaluate the potential metastatic sites. Magnetic resonance imaging (MRI) of the brain is used in patients with stage IV, optional in stage III and not used in patients with stage I and II disease. The diagnosis of metastases is evaluated by Positron emission tomography (PET)/CT. This technique complements conventional CT/MRI imaging in the staging of patients who have solitary or oligometastatic disease where surgical resection is most relevant. The lesions suspected of cutaneous melanoma are subjected to dermoscopic examination and if dermatologist deems it necessary, evaluated with excisional biopsy. The histological examination allows to decide whether to perform a further surgical excision and an SNLB; after a correct melanoma staging to decide the subsequent treatment (19, 20). Therefore after the excision of the lesion and histologic evaluation it is mandatory to perform a correct staging to decide whether a further surgical excision should be performed. Ultrasonography is widely used in medicine (21–23). In recent years, US and especially HFUS have become popular among dermatologists. Skin US offers essential information for the diagnosis, therapeutical management, and follow-up of tumoral and non-tumoral cutaneous pathology. It seems that HFUS examination may be useful in pre-operative evaluation of CM, and it may correlate with histology (24). Modern HFUS equipment allows highly accurate visualization of the skin layers and appendages up to histological details (25–28). Probes ranged from 15 to 22 MHz allowed visualization of the epidermis and dermis, including adjacent tissues 1 to 2 cm deep from the basal dermal layer (16).

Moreover ultra-HFUS has ultrasound frequencies higher than 30 MHz, which allow to obtain submillimeter resolution of superficial anatomical structures (29).

The image quality is influenced by the resolution, the key element in measuring the thickness and depth of skin changes (30). The typical ultrasound image of healthy skin is composed of three elements: epidermis, also known as epidermal echo, dermis and subcutaneous tissue (30).

HFUS cannot detect pigments such as melanin but allows a non-invasive evaluation of the primary tumor. It is already able to calculate a Breslow index in a large number of patients with CM (1).

Many literature studies provide US information on primary skin melanoma lesions (30–32). The first US evaluations were performed with 14 MHz probes. The 20-MHz probe was used in five studies, it has an axial resolution that goes from 50 to 80 µm and lateral resolutions to 100 µm in Bessoud et al., 2003, Clement et al., 2001, Lassau et al., 1997 and Rallan et al., 2007 at 300 µm in Harland et al., 2000 (12, 33–36).

As far as these studies are concerned, it remains unclear how the authors obtained the resolution values. Some parameters such as dynamic signal range and signal-to-noise ratio were not reported in the studies, and more often the diagnostic information provided on the lesions appeared to be poorly detailed (37).

Bessoud et al., 2003 evaluated with HFUS 130 pigmented lesions and added a Color Doppler study in 107 lesions. Ultrasound features were linked with anatomo-pathological specimen. Of these lesions: 57% invasive melanoma, 29% benign nevi, 4% basal cell carcinoma (BCC), 4% seborrheic keratosis and other benign lesions (32, 34).

Lassau et al., 1997 evaluated 70 skin lesions, clinically suspected of CM (35) and of BCC (32). HFUS and color Doppler were performed for each lesion, only eight lesions of these were not visualized and therefore excluded. Of these lesions 19 (27%) were invasive melanoma, 31 (44%) BCC, one neurosarcoma, and 12 (17%)were benign nevi (3 of the seven lesions not visualized on HFUS were melanomas) (12). In both studies, the sensitivity of the combined characteristics of HFUS was 100% with a specificity of 33% (95% CI 20% to 48%) in Bessoud et al, 2003 (130 lesions; 65 melanomas) and 73% (95% CI 57% to 85%) in Lassau et al., 1997 (62 lesions; 19 melanomas) (the lower limits of the 95% CIs for sensitivity were 94% and 82%, respectively).

Lassau et al., 1997 determined a specificity of 8% (95% CI 0% to 36%) on 32 lesions, 19 of which were melanomas. Both studies have not visualized five melanomas in the US (38).

Lassau et al., 1997 who evaluated the hypoechoic, homogeneous, well-defined and vascularized lesions, saw that there is no difference in the sensitivity and specificity achieved using HFUS alone for the discrimination of invasive melanoma (n = 19) from all other included lesions (n = 44) (39).

The HFUS and Doppler features can be combined according to both Bessoud et al., 2003 and Lassau et al., 1997, sensitivities were 34% (95% CI 22% to 47%; n = 65 melanomas) and 16% (95% CI 3% to 40%; n = 19 melanomas) with 100% specificity (95% CI 92% to 100%) respectively for both studies (n = 45 and n = 44).

Harland et al., 2000 and Rallan et al., 2007 reported quantitative assessments of the US image evaluating the acoustic differences between common pigmented lesions.

Both studies included only melanoma, melanoma in situ, benign naevi, or seborrheic keratosis (n = 19, 6, 15, 29 in Harland et al., 2000; and n =14, 11, 38, 24 in Rallan et al., 2007).

Harland et al., 2000 compared melanoma and seborrheic keratosis (benign naevi excluded) (35, 36).

Rallan et al., 2007’s work on a prototype 3D HFUS C-scan with “reflex transmission” imaging found significant differences in the mean values between melanoma and seborrheic keratosis and between melanoma and benign naevi (39).

Kaikaris et al., 2011 described the use of HFUS (14 MHz) and the association between US and morphological findings in measuring melanoma thickness.

They found a low US correlation between the Breslow index for thin melanomas (1–2 mm) and a significant correlation for thicker melanomas (> 2 mm). Measurements made with ultra-HFUS (20 MHz) were found to be well correlated with the depth of thick melanomas but were not accurate enough for thinner melanomas.

Evidence suggests that HFUS (20 MHz) may be the best tool for the estimations of tumor volume more than 2D-US (40). The first significant US reports of melanoma were performed using fixed HF probes ranging from 20 to 100 MHz.

Solivetti et al., 2014, define the HFUS as a useful technique for the detection of melanoma in-transit metastases (41). This study was performed on 600 patients with melanoma (thickness> 1 mm) resulted negative to objective examination at clinical follow-up; the US detected in-transit metastases in 63 patients with a total of 95 lesions (41). All these lesions have not reported false positive or false negative (41).

Botar et al., 2015 document the positive correlation between the Breslow index with the involvement of the lymph nodes and risk of distant metastasis. This study performed the characterization of the lesion with elastography but used the 40-MHz probe for the semiquantitative analysis. The information obtained with HFUS showed a good correlation between sonometry and histometry (r = 0.88), with an average difference of 0.39 mm (relative difference 28%) (35, 42). Tumors with a thickness between 0.55 and 0.95 mm were found to be incorrectly classified according to histology in 34%, and tumors with a thickness between 1.30 and 1.70 mm were classified incorrectly in 50% of cases. These last results are due to the low penetration of ultrasound with fixed frequency equipment (about 6 mm at 20 MHz, 3 mm at 75 MHz, and 1 mm at 100 MHz).

On the other hand, probes with variable frequency from 10 to 15 MHz and multi-channeled color Doppler evaluation allow differentiating melanomas measuring < o > 1 mm in thickness (43). This evaluation is essential in choosing to perform an SNL biopsy, which is indicated in melanomas measuring more than 1 mm in thickness (42).

Gambichler et al., found an almost similar relationship to histology, with a correlation coefficient of 0.99 with both 20- and 100-MHz transducers (44).The use of 100 MHz was more accurate than the 20 MHz. They included only lesions ≤ 1 mm thick, limiting the evaluation of lesions> 1 mm thick. Machet et al., Gambichler et al., and Pellacani et al., found that the US measurements were slightly overestimated compared to the histological size but concluded that US has a strength correlation with melanoma thickness (10, 45, 46).

For the first time, Reginelli et al., described the HFUS analysis of the CM using probes ranged from 50 to 70 MHz. In this study 14 CM have been analyzed. They present oval aspects and a fusiform shape, inhomogeneous, hypoechoic, smooth edges, and variable vascularization (1, 47, 48).

After several studies on small animals, the first HFUS for clinical use could be introduced for clinical use. The availability to use HF between 50 and 70 MHz is much higher than the conventional US systems, providing a resolution up to 30 microns and a penetration of about 15 mm (1). They considered the US performed with HF probes more accurate because the result corresponds to *in vivo* tissue without dehydration or fixation. The thickness obtained from US evaluation was compared to that obtained on the biopsy piece, and a favorable agreement was seen with the Breslow thickness (39, 49–51).

## Conclusions

The application of ultrasound to dermatology is becoming more and more frequent. The ultrasound examination offers significant advantages and being it minimally invasive it is easily repeatable. In particular, the use of equipment with high-frequency probes provides important information, especially in the pre-operative, thus allowing a broader diagnostic-therapeutic evaluation, as well as later follow-up.

## Author Contributions

All the authors contributed equally to this work. All authors contributed to the article and approved the submitted version.

## Funding

This study was funded by “Program Valere” by University of Campania “Luifi Vanvitelli” (Grant No: DR 05 - 04/01/2018), Naples, Italy.

## Conflict of Interest

The authors declare that the research was conducted in the absence of any commercial or financial relationships that could be construed as a potential conflict of interest.
